# Water-induced strong protection against acute exposure to low subzero temperature of adult *Aedes albopictus*

**DOI:** 10.1371/journal.pntd.0007139

**Published:** 2019-02-04

**Authors:** Meichun Zhang, Dongjing Zhang, Yongjun Li, Qiang Sun, Qin Li, Yali Fan, Yu Wu, Zhiyong Xi, Xiaoying Zheng

**Affiliations:** 1 Sun Yat-sen University—Michigan State University Joint Center of Vector Control for Tropical Diseases, Zhongshan School of Medicine, Sun Yat-sen University, Guangzhou, China; 2 Key Laboratory for Tropical Disease Control, Ministry of Education, Sun Yat-sen University, Guangzhou, China; 3 Guangdong Provincial Engineering Technology Research Center for Diseases-Vectors Control, Sun Yat-sen University, Guangzhou, China; 4 Section of Pulmonary, Critical Care and Sleep Medicine, Department of Internal Medicine, Yale School of Medicine, New Haven, CT, United States of America; 5 Department of Microbiology and Molecular Genetics, Michigan State University, East Lansing, MI, United States of America; Fundaçao Oswaldo Cruz, BRAZIL

## Abstract

As an important vector of dengue and Zika, *Aedes albopictus* has been the fastest spreading invasive mosquitoes in the world over the last 3–4 decades. Cold tolerance is important for survival and expansion of insects. *Ae*. *albopictus* adults are generally considered to be cold-intolerant that cannot survive at subzero temperature. However, we found that *Ae*. *albopictus* could survive for several hours’ exposure to -9 to -19 ^o^C so long as it was exposed with water. Median lethal time (LT_50_) of *Ae*. *albopictus* exposed to -15 and -19 ^o^C with water increased by more than 100 times compared to those exposed to the same subzero temperature without water. This phenomenon also existed in adult *Aedes aegypti* and *Culex quinquefasciatus*. *Ae*. *albopictus* female adults which exposed to low subzero temperature at -9 ^o^C with water had similar longevity and reproductive capacity to those of females without cold exposure. Cold exposure after a blood meal also have no detrimental impact on survival capacity of female adult *Ae*. *albopictus* compared with those cold exposed without a blood meal. Moreover, our results showed that rapid cold hardening (RCH) was induced in *Ae*. *albopictus* during exposing to low subzero temperature with water. Both the RCH and the relative high subzero temperature of water immediate after cold exposure might provide this strong protection against low subzero temperature. The molecular basis of water-induced protection for *Ae*. *albopictus* might refer to the increased glycerol during cold exposure, as well as the increased glucose and hsp70 during recovery from cold exposure. Our results suggested that the water-induced strong protection against acute decrease of air temperature for adult mosquitoes might be important for the survival and rapid expansion of *Ae*. *albopictus*.

## Author summary

*Aedes albopictus* is one of two most important vectors for dengue and zika. During the last 3–4 decades, this mosquito has spread from native Asian area to all continents except Antarctica, becoming the most invasive mosquitoes which imposed extensive public health threat to human beings throughout the world. Cold tolerance is important for distribution and survival of insects. During the expansion of *Ae*. *albopictus*, especially a spatial expansion to cooler climate areas, it needs to cope with cold temperatures. Moreover, because of such widespread distribution adult *Ae*. *albopictus* will certainly often encounter sudden drops in air temperature even below subzero that often happens in early spring and winter, and late autumn. Thus far, adult *Ae*. *albopictus* are generally considered to be cold-intolerant that can not survive at subzero temperature. In this study, we found that water can provide strong protection against low subzero temperature even below -10 ^o^C. Cold exposure of adult female *Ae*. *albopictu*s to low subzero temperature with water either before or after a blood meal have no detrimental impact on fitness costs of these adult mosquitoes. Considering water is common in nature, our results indicated that during the expansion of *Ae*. *albopictus* especially when adult mosquitoes encounter a sudden drop in air temperature water could be a good shelter for cope with such cold temperature below subzero.

## Introduction

Cold tolerance or cold hardiness, the ability of an insect to survive at low temperature, is important in defining the distribution and survival of insects. There are two different cold hardening in insects at present. One is accomplished by long term (weeks or months) cold acclimatization to overwinter that occurs in an inactive or diapausing stage; the other called rapid cold hardening (RCH) which is accomplished by a brief exposure (minutes or hours) to low temperature that occurs even in feeding and reproductive stages [1–4]. As an efficient ability utilized by insects to survival in environment with rapid and unexpected changes in temperature, RCH has been found in numerous insect species belonging to different orders including Diptera [2, 4–7].

*Aedes albopictus* is an epidemiologically important vector for several arboviruses such as dengue, yellow fever, zika, and chikungunya. During the last 3–4 decades, *Ae*. *albopictus* has spread from native Asian area to all continents except Antarctica, becoming the most invasive mosquitoes which imposed extensive public health threat to human beings throughout the world [8–12]. During the spread of this species, a spatial expansion to cooler climate areas has also been reported and the ability to rapidly produce low temperature phenotypes has been considered as an important factor for the successful establishment in these cooler habitats [13]. Therefore, cold hardiness is a key trait for the distribution of this species and strong resistance to cold temperature provides more chances for efficient invasiveness to colder zones.

Adult *Ae*. *albopictus* are generally considered to be cold-intolerant that can not survive at subzero temperature and the only life stage that can cope with this low temperature is its eggs [13, 14]. Thus, the studies on the cold hardiness of this mosquito have been focused on eggs [13–19]. Moreover, the RCH has not been reported in adult and eggs of this species yet. In our current study, we found that adult *Ae*. *albopictus* could survive for several hours’ exposure under -10 ^o^C when transferred from room temperature to low subzero temperature with water, which was also found in adult *Aedes aegypti* and *Culex quinquefasciatus*. Median lethal time (LT_50_) of adult mosquitoes was increased by nearly 100 times compared to those transferred directly to subzero temperature lower than -10 ^o^C without water. This indicated that RCH was also existed in adult mosquitoes and that water in nature might provide strong protection for adult mosquitoes against sudden drop in air temperature that often happens in early spring and winter, and late autumn [2, 4, 6, 7]. The aim of this study is to compare the cold hardiness of adult *Ae*. *albopictus* exposed to low subzero temperature with water with those exposed directly, analyze the possible molecular mechanism for this cold hardiness and determine the impact of exposure to low subzero temperature with water on fitness costs of adult female *Ae*. *albopictus*.

## Materials and methods

### Mosquito strain and rearing

Mosquitoes used in this study including *Ae*. *albopictus*, *Ae*. *aegypti* and *Cx*. *quinquefasciatus* were all established for several years in our laboratory with Guangdong origin. All mosquitoes were reared in a climate-controlled room at 28 ± 1°C and 80 ± 5% relative humidity with a 12:12-hour (light: dark) photoperiod. Adult mosquitoes were provided with 10% glucose solution.

### Subzero temperature exposure

To evaluate the survivorship, 3 groups of 20 adult mosquitoes with 3- to 5-day-old were immobilized by CO_2_ and then transferred to a disposable 100ml-plastic cup with (treatment) or without (control) dechlorinated tap water (50 mL). A plastic lid was used to cover with cup for preventing escape of mosquitoes during experiment. These adults were then allowed to recover from anaesthetization at room temperature for 1 h. Subsequently, the cups of mosquitoes with water were exposed to low subzero temperatures at -9, -15 and -19 ^o^C for 3 to 8 h, and the cups of mosquitoes without water were exposed to the same temperatures for 3 to 30 min. Twenty adults (one cup) were removed from each temperature at a 1 h interval for cups with water and 1- to 5-min interval for cups without water until 100% mortality were attained. The exposed mosquitoes were then transferred to 650ml-plastic cages with a piece of wet filter paper to keep humidity and maintained at normal climate-controlled room. The mosquito survival was recorded 24 h after cold exposure and survival was defined as the ability of righting themselves and flies [20].

### Fitness determinants

To evaluate the impact of cold exposed to low subzero temperature on fitness costs of female adult *Ae*. *albopictus*, 3 groups of 30 adult mosquitoes with 3- to 5-day-old were cold exposed to -9 ^o^C with water for 3 to 5 h as described above. Subsequently, the exposed mosquitoes were transferred to cages, maintained under normal climate-controlled room and provided with 10% glucose solution. Control groups of 90 female mosquitoes without cold exposure were also transferred to cages and maintained under normal condition. Three days after recovery from cold exposure dead mosquitoes were discarded and survived mosquitoes that have starved for 24 h were blood fed on mice for 30 min. The engorged mosquitoes were then counted and transferred to new cages, and were aspirated into individual 50 mL Corning tubes 2 days post-blood meal (PBM) with bottom lining of moist filter paper supported by water-soaked cotton [21]. Two days after oviposition, mosquitoes were aspirated out and killed by cold, eggs were removed out for maturation for 3 days and then counted. After maturation, eggs on filter paper were immersed in 50 mL water in a 100ml-plastic cup and egg hatch rates were determined by counting the number of hatched second instar larvae [22]. Adult lifespan of female *Ae*. *albopictus* after cold exposure to -9 ^o^C with water for 3 h were also monitored and compared with control group without cold exposure. Cold treatments were conducted as above and dead mosquitoes were removed at 24 h after recovery. Survived mosquitoes were reared under normal condition and dead mosquitoes were counted and removed daily for a month.

To evaluate the impact of cold exposure on blood-fed *Ae*. *albopictus*, engorged mosquitoes were collected and transferred to a new cage. Three groups of 30 adult mosquitoes were selected at 24 and 48 h PBM and exposed to -9 ^o^C with water for 3 h. Mosquitoes without a blood meal were also selected and cold exposed at the meanwhile. Cold exposed mosquitoes were maintained in cages under normal condition and survivals were recorded 24 h after recovery. Two days after blood meal, mosquitoes of cold exposure at 24 h PBM were reared individually, and mosquitoes of cold exposure at 48 h PBM were reared as a pool, egg numbers and egg hatch rates were determined as above.

### Water temperature monitoring

After transferring water from room temperature into low subzero temperature, the dynamics of water temperature was monitored by a mini-thermometer (Testo 175 H1, Lenzkirch, Germany). The temperature probe was wrapped by plastic film and immersed into 50 mL water in a disposable 100ml-plastic cup and then transferred to different low subzero temperature for 12 h. Temperature was detected and recorded at a 5-min interval.

### Glycerol and glucose analysis

Adult mosquitoes were cold exposed as above and collected at 0, 1, 2 and 4 h after recovery from cold treatment. Six adults were pooled in a single replicate and five replicate biological assays were performed. These sampled mosquitoes were homogenated in 300 μL distilled deionized water. Two hundred microlitres of the homogenates were used for RNA extraction and another 100 μL were filtered at room temperature through a spin filter (Pall, nanosep 10k Omega, NY) at 12,000 rpm for 15 min. For glycerol analysis, filtered homogenates were diluted 1:100 v/v with distilled deionized water. Next, 10 μL of the diluted homogenates were incubated with 100 μL Master Reaction Mix (Sigma-Aldrich, MAK117, USA) for 20 min at room temperature. Absorbance was measured at 570 nm and glycerol contents were calculated from standard curve. Glucose levels were determined by using the Glucose (GO) Assay Kit (Sigma-Aldrich, GAGO-20, USA) according to the manufacturer’s protocol with minor modifications. The above filtered homogenates were diluted 1:5 v/v with distilled deionized water and 50 μL of these diluted samples were then incubated with 100 μL Assay Reagent for 30 min at 37 ^o^C. After the incubation, 100 μL of 12N H_2_SO_4_ were added to stop the reaction. Then, the absorbance was measured at 540 nm and glucose contents were calculated from standard curve.

### Real-time PCR

Total RNA was extracted from samples collected above using TRIzol Reagent (Invitrogen, Carlsbad, CA) and the first strand cDNA was synthesized using HiScript II Q SuperMix for qPCR (+dDNA wiper) (Vazyme, Nanjing, China) following the manufacturer's protocol. Relative expression level of Hsp70 mRNA was performed by quantitative real-time PCR (qPCR) on the LightCycler96 Detection System (Roche, Mannheim, Germany) using TB Green Premix Ex Taq II (Tli RNaseH Plus) (TaKaRa, Otsu Shiga, Japan). The primer sequences for Hsp70 were forward (5’-TACCAACGGCGACACTCAC-3’) and reverse (5’-TTGCGGATGTCCTTACCCT-3’). Each reaction consisted 0.5 μL of cDNA, forward and reverse primer (10 μM), 10 μL of TB Green Premix Ex Taq II (2×), and 8.5 μL of distilled deionized water to a final volume of 20 μL. The qPCR program was 95 ^o^C for 30 sec, then 40 cycles of 95°C for 5 sec and 60°C for 30 sec followed by a melt-curve analysis. *Ae*. *albopictus* rpS7 was used as internal control and the relative Hsp70 expression of cold exposed samples were calibrated by samples collected at room temperature that without cold treatment. The relative expression levels of Hsp70 were determined by using the 2^-△△CT^ calculation method [23].

### Statistical analyses

All data were analyzed by using SPSS statistics 19. Comparison of LT_50_, egg numbers and egg hatch rates per female (except egg hatch rate of mosquitoes cold exposed at 48h PBM) between different groups were conducted using Student’s *t*-test. Comparison of the percent of mosquitoes imbided a blood meal between cold exposed and non-exposed groups, of the survival of mosquitoes cold exposed at 24 h PBM with those without blood meal and of the egg hatch rate of mosquitoes cold exposed at 48 h PBM with those maintained at room temperature were performed using chi-square test. Comparisons of glycerol, glucose and hsp70 mRNA levels between groups were assessed using ANOVA followed by Tukey’s multiple comparison.

### Accession number

The GenBank accession number of hsp70 mentioned in the text is JN132155.1.

## Results

### Survival of adult mosquitoes experienced acute exposure to low subzero temperature with water

**Fig 1:**
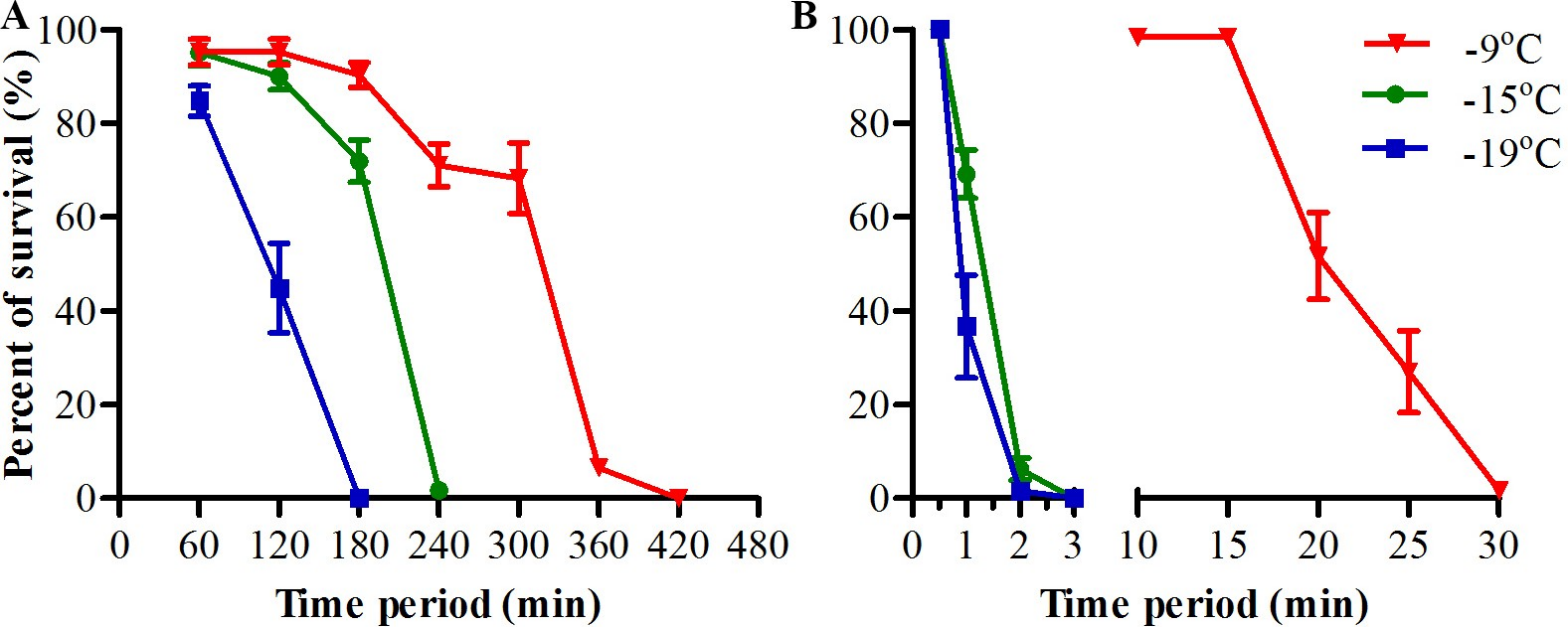
Survival of adult female *Ae*. *albopictus* after exposure to different subzero temperature. Adult female *Ae*. *albopictus* were transferred from room temperature to different subzero temperature with (A) or without water (B). Adult mosquitoes were removed (at 1-h interval for mosquitoes exposing with water or at 1- or 5-min interval for mosquitoes exposing without water) from subzero temperature and recovered under normal rearing condition until 100% mortality were reached. N = 3 groups of 20 adult mosquitoes for each data point.

**Fig 2:**
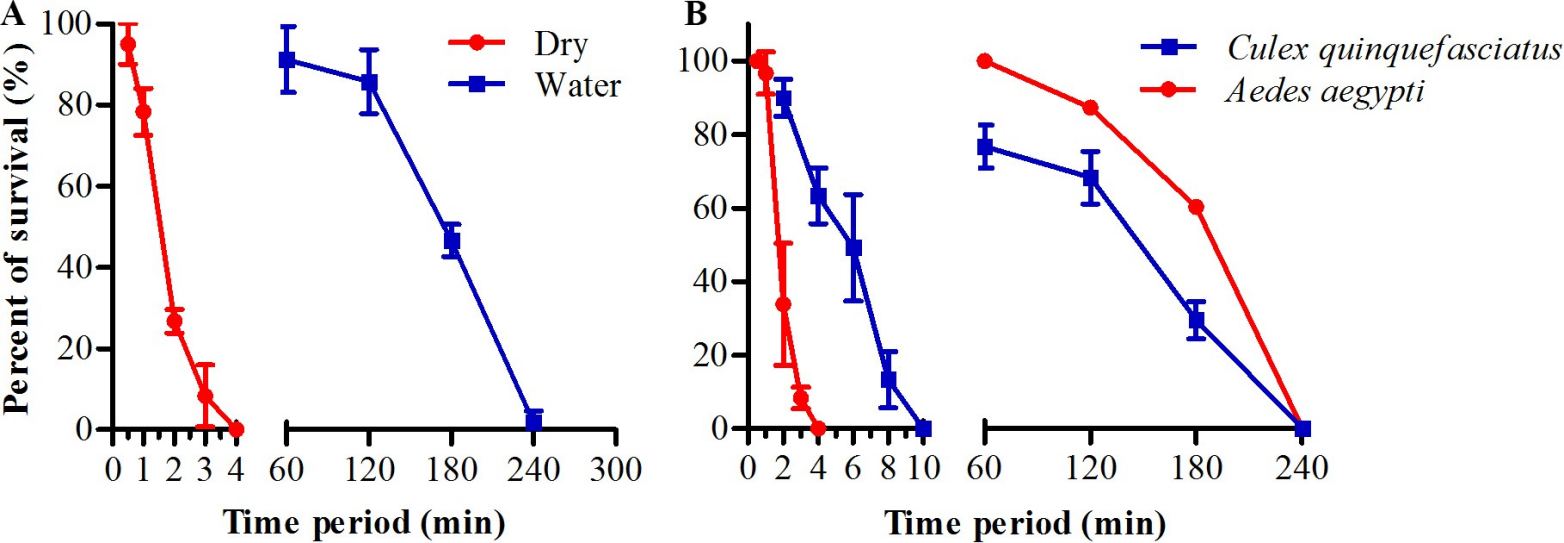
Survival of adult mosquitoes after exposure to low subzero temperature. Adult male *Ae*. *albopictus* (A) and female *Ae*. *aegypti* and *Cx*. *quinquefasciatus* (B) were transferred from room temperature to -15 ^o^C with (right of A and B) or without water (left of A and B). Adult mosquitoes were removed (at 1-h interval for mosquitoes exposing with water or at 1- or 2-min interval for mosquitoes exposing without water) from subzero temperature and recovered under normal rearing condition until 100% mortality were reached. N = 3 groups of 20 adult mosquitoes for each data point.

We found that adult *Ae*. *albopictus* could survive for several hours’ exposure to subzero temperature even below -10 ^o^C when it was transferred from room temperature to the low temperature with water. Subsequently, we analyzed the survival of adult female *Ae*. *albopictus* that transferred to different low subzero temperatures from -9 ^o^C to nearly -20 ^o^C with water and compared to the mosquitoes that directly transferred to the same temperatures without water. The results showed that when exposed adult mosquitoes to low subzero temperature with water the cold tolerance of these mosquitoes were strongly increased compared to those directly exposed without water (Fig 1). About 70 to 45% of these mosquitoes survived a 5 to 2 hours’ exposure to -9 to -19 ^o^C when exposed with water. However, mosquitoes that directly transferred to the same low temperature without water were found to 100% mortality within 3 to 30 min. The LT_50_ of these adult *Ae*. *albopictus* were about 292, 184 and 106 min for those exposed to -9, -15 and -19 ^o^C with water, respectively, and these were 13.6, 145.1 and 108.7 times longer than those exposed to the same low temperature without water (Table 1). Moreover, we did the same analysis on adult male *Ae*. *albopictus*, female *Ae*. *aegypti* and *Cx*. *quinquefasciatus* to see if the phenomena also existed in male and other mosquitoes. The results suggested that when transferred these adult mosquitoes from room temperature to low subzero temperature (-15 ^o^C) with water the cold tolerance were also significantly increased compared to those transferred to the same low temperature without water (Fig 2 and Table 2).

**Table 1:** Lethal time of 50% of adult female *Aedes albopictus* after exposure to different low temperature.

Temperature (oC)	LT_50_ (min)		Fold increase (Water/Dry)	*P-value	^§^P-value
	Dry	Water			
**-9 (-2.0^#^)**	21.4 ± 1.58 (85.4 ± 1.39^¶^)	292.2 ± 6.56	13.6* (2.7^§^)	< 5.0×10–7	< 1.0×10–6
**-15 (-3.0)**	1.27 ± 0.17 (57.2 ± 2.78)	184.3 ± 2.06	145.1 (2.3)	< 5.0×10–5	< 5.0×10–7
**-19oC (-5.0)**	0.98 ± 0.15 (38.3 ± 2.03)	106.5 ± 7.89	108.7 (2.0)	< 5.0×10–5	< 0.0005

^#^ The relative high subzero temperature of water immediate after decreased below subzero when transferring to corresponding low subzero temperature.

^¶^ The LT_50_ of mosquitoes after exposure to corresponding high subzero temperature.

* Fold increase in LT_50_ of mosquitoes after exposure to low subzero temperature with water compared to without water.

^§^ Fold increase in LT_50_ of mosquitoes after exposure to low subzero temperature with water compared to those exposed to the corresponding high subzero temperature without water (Time needed for decrease in temperature of water after cold exposure to subzero was subtracted).

**Table 2:** Lethal time of 50% of adult mosquitoes after exposure to -15°C.

Mosquitoes	LT_50_ (min)		Fold increase (Water/Dry)	P-value
	Dry	Water		
**Male *Aedes albopictus***	1.65 ± 0.14	162.9 ± 6.83	98.7	< 5.0×10^−6^
***Aedes aegypti***	1.96 ± 0.04	176.7 ± 2.75	90.2	< 5.0×10^−8^
***Culex quinquefasciatus***	5.25 ± 0.14	132.0 ± 3.54	25.1	< 5.0×10^−7^

### A rapid cold-hardening response of adult *Ae*. *albopictus* was induced when exposed to low subzero temperature with water

**Fig 3:**
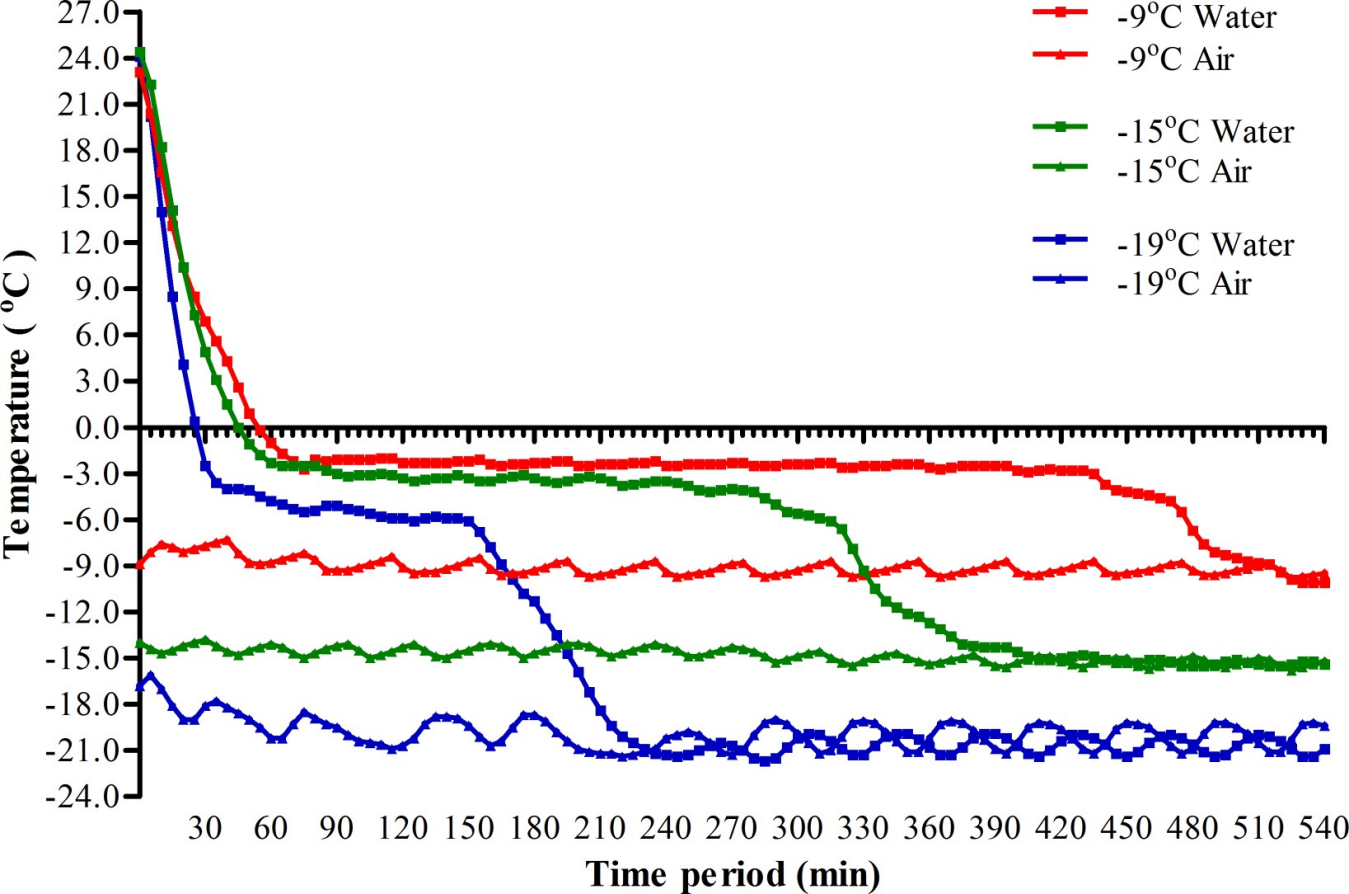
The change of water temperature after transferring from room temperature to different low subzero temperature. The temperatures of water were monitored by a mini-thermometer at 5-min interval. The mini-thermometer with ice was removed from subzero temperature at 12h after exposure and the ice was naturally melted.

**Fig 4:**
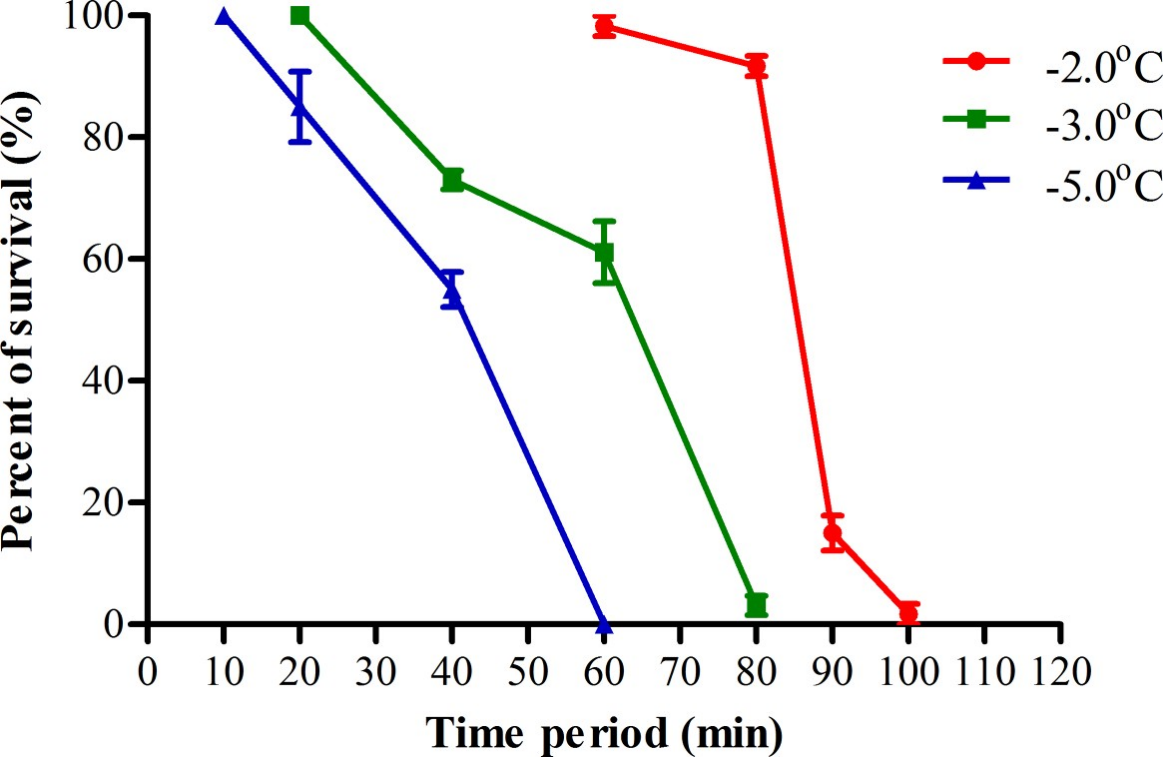
Survival of adult female *Ae*. *albopictus* after exposure to high subzero temperature without water. Adult female *Ae*. *albopictus* were transferred from room temperature to different high subzero temperatures without water. Adult mosquitoes were removed at 10-min interval from subzero temperature and recovered under normal rearing condition until 100% mortality were reached. N = 3 groups of 20 adult mosquitoes for each data point.

When transferring from room temperature to low subzero temperature with water adult mosquitoes will fall on the surface of water after anaesthetized by cold, we detected the change in temperature of water during this process (Fig 3). We found that when transferring from room temperature to -9, -15 and -19 ^o^C, the water temperature would reach subzero and iced within 55, 45 and 25 min, respectively. After that the temperature of ice kept at a relative high level of -2, -3 and -5°C for 6, 3 and 2 h, respectively, and then rapidly decreased to the level equal to the low ambient air temperature. To evaluate whether it is the relative high temperature of ice just after dropping under subzero that protected adult mosquitoes from low temperature, we analyzed the survival of adult female *Ae*. *albopictus* when exposed to these relative high subzero temperature without water (Fig 4 and Table 1 in parentheses). The results indicated that when exposed to -2 to -5°C, 100% mortality of these adult mosquitoes were reached within 60 to 100 min and that the LT_50_ were about 85, 57 and 38 min for those exposed to -2, -3 and -5 ^o^C, respectively. Even subtracting the time needed to cool water from room temperature to the relative high subzero temperature, the LT_50_ of adult female *Ae*. *albopictus* transferred to -9 to -19 ^o^C with water still were 2.7 to 2.0 times longer than those directly exposed to -2 to -5°C without water (Table 1). These results indicated that when exposed adult mosquitoes to low subzero temperature with water a RCH response was induced during this process.

### Fitness costs of female *Ae*. *albopictus* exposed to low subzero temperature with water

**Fig 5:**
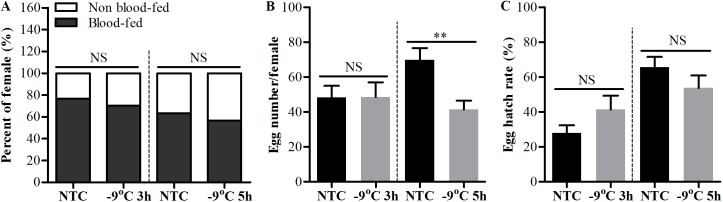
Fitness costs of female *Ae*. *albopictus* exposed to low subzero temperature with water. Adult female *Ae*. *albopictus* were transferred from room temperature to -9 ^o^C with water and removed after 3 and 5 hours’ exposure. Adult mosquitoes were recovered in cages under normal rearing condition. Live mosquitoes were fed on mice for about 30 min. NTC were mosquitoes maintained under normal rearing condition and fed on a blood meal. The number of mosquitoes that imbided a blood meal (A), mean egg number (B) oviposited by and mean egg hatch rate (C) from individual female mosquitoes. Statistical differences between the numbers of blood-fed mosquitoes were determined by chi-square test and between egg numbers, egg hatch rates of individual female mosquito were determined by Student’s *t*-test. Error bars indicate SEM. **P<0.01; NS, not significant. N = 3 groups of 30 adult mosquitoes were cold exposed and 30 blood-fed mosquitoes were individually reared and laid eggs.

**Fig 6:**
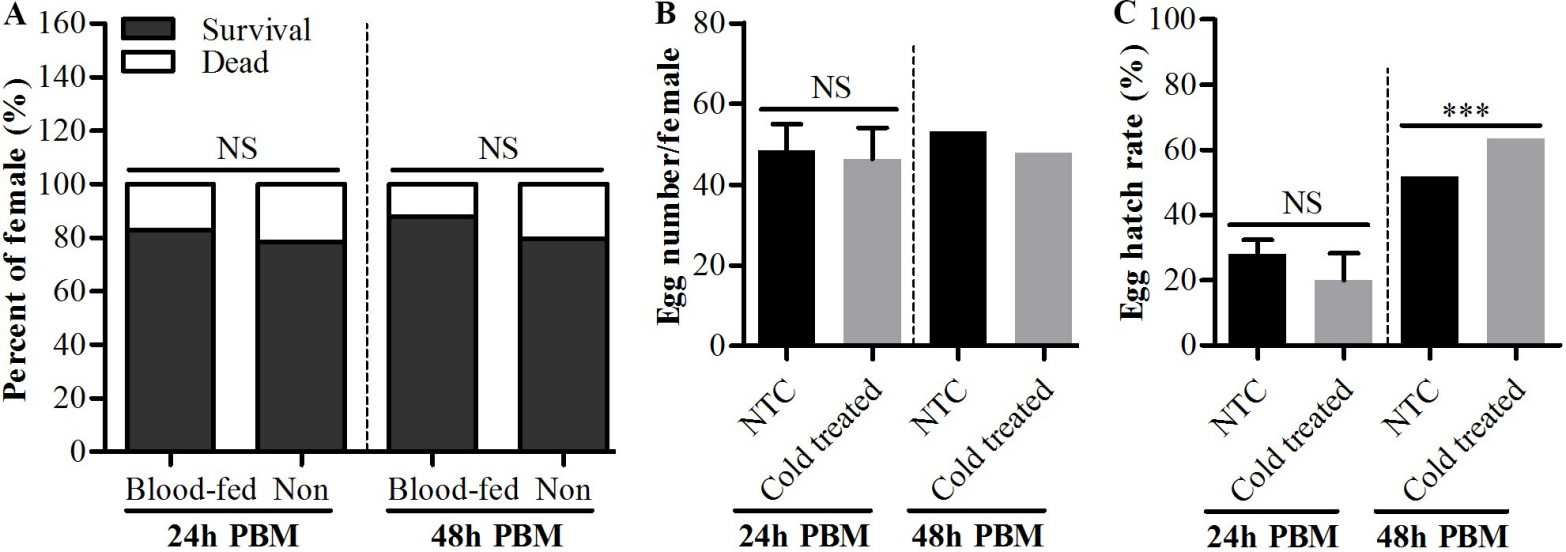
The impact of exposing to low subzero temperature with water on survival, fecundity and fertility of female *Ae*. *albopictus* after a blood meal. Adult female *Ae*. *albopictus* were transferred from room temperature to -9 ^o^C with water at 24 and 48h PBM. The mosquitoes were recovered in cages under normal rearing condition after 3 hours’ cold exposure. Groups of mosquitoes without blood meal were also cold exposed as above. (A) The survival of mosquitoes were recorded 24h after recovery. The egg numbers (B) were counted 2 days after oviposition and egg hatch rate (C) were determined at second instar larvae. Statistical differences between the survivals of different groups, egg hatch rates between cold treated mosquitoes at 48h PBM and not treated mosquitoes (NTC) were determined by chi-square test, and between egg numbers of cold treated mosquitoes at 24h PBM and NTC were determined by Student’s *t*-test. The NTC groups that compared with cold treated mosquitoes at 24h PBM of (B) and (C) were the same as NTC groups from Fig 5 that compared with groups of -9 ^o^C 3h. Error bars indicate SEM. ***P<0.0001; NS, not significant. N = 3 groups of 30 blood-fed and non blood-fed mosquitoes were cold exposed and 30 blood-fed mosquitoes were individually reared and laid eggs.

Since adult *Ae*. *albopictus* could survive several hours’ exposure to low subzero temperature with water, it is important to know the effects of cold exposure on the bite behavior and reproductive capacity of female adults. Our results showed that after exposure to -9°C for 3 and 5 h with water, there still were 70.2% and 56.7% of mosquitoes that successfully had a blood meal on mice, respectively, and there was no significant difference between cold exposed and non-exposed mosquitoes (Fig 5A). The fecundity (egg numbers per female) and egg hatch rates between cold exposed and non-exposed mosquitoes also had no significant difference except for the fecundity of mosquitoes exposed to -9°C for 5 h (Fig 5B and 5C). Although the fecundity of these mosquitoes was significantly lower than the mosquitoes without cold exposure, they still could lay about 41 eggs per female after 5 hours’ exposure to -9°C with water. Meanwhile, we found that blood meal had no impact on cold tolerance of adult female mosquitoes compared to mosquitoes without blood meal. There were no significant difference on survival capacity between blood-fed and non blood-fed mosquitoes after 3 hours’ exposure to -9°C with water at both 24 and 48h PBM (Fig 6A), and these mosquitoes could still lay viable eggs (Fig 6B and 6C). Indeed, the egg hatch rate of mosquitoes cold exposed at 48h PBM was significantly higher than mosquitoes without cold exposure after blood meal (Fig 6C). Moreover, the lifespan of adult female *Ae*. *albopictus* was also compared between cold exposure and no exposure, and no significant difference was observed (S1 Fig).

### Glycerol and glucose levels in adult *Ae*. *albopictus* exposed to low subzero temperature

**Fig 7:**
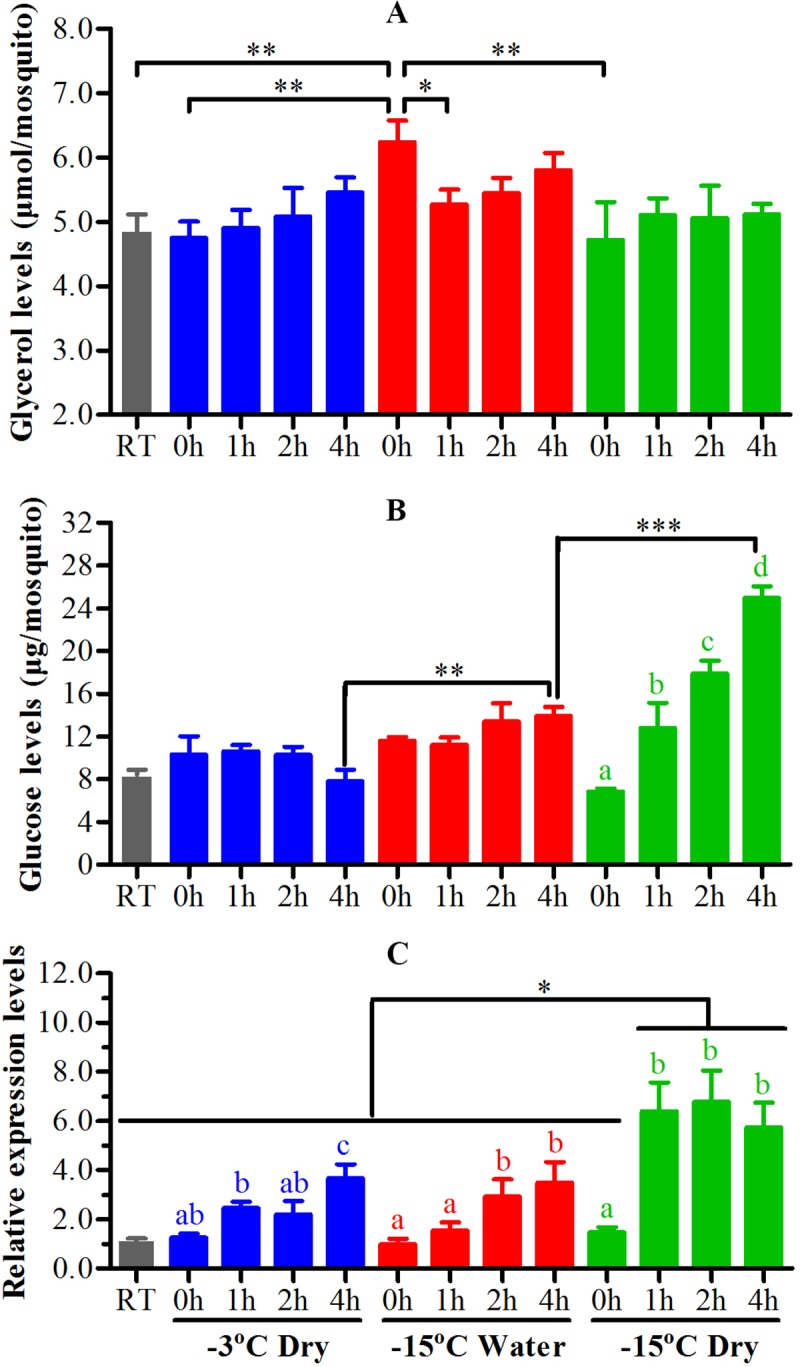
The change of glycerol and glucose levels and the expression of Hsp70 mRNA in adult female *Ae*. *albopictus* after exposure to different subzero temperature. Adult female *Ae*. *albopictus* were transferred from room temperature to -3 ^o^C without water (blue) and -15 ^o^C with (red) or without water (green). Adult mosquitoes were then removed from subzero temperature after 90 min for exposure to -3 ^o^C, 3 min for exposure to -15 ^o^C without water and 160 min for exposure to -15 ^o^C with water and recovered under normal rearing condition. The contents of glycerol (A) and glucose (B) and the expression levels of hsp70 mRNA (C) were analyzed at 0 to 4h after recovery from cold exposure. The data shown are means ± SEM of five replicates with six whole mosquitoes for each. Columns that do not share the same letter are significantly different from each other within each group. The differences in glycerol and glucose contents and hsp70 mRNA levels were assessed by ANOVA followed by Tukey’s multiple comparison. *P < 0.05, **P < 0.01, ***P < 0.001.

Since the cold tolerance was significantly increased when exposed adult mosquitoes to low subzero temperature with water, we analyzed the levels of two important cryoprotectants glycerol and glucose in whole body of adult *Ae*. *albopictus* that exposed to -15 ^o^C with water for 2.7 h and compared to those exposed to -3 and -15 ^o^C directly for 1.5 h and 3 min, respectively. The duration of time were chose because at this duration mosquitoes exposed to -3 and -15 ^o^C directly were 100% mortality but mosquitoes exposed to -15 ^o^C with water were less than 30% mortality at the same duration of time as those exposed to -3 ^o^C directly after subtracting the time needed to cool water from room temperature to -3 ^o^C. The results showed that the glycerol level of mosquitoes exposed to low temperature with water was 6.2 μmol per mosquito just after recovery from cold exposure and this was significantly higher than those directly exposed without water and those maintained at room temperature (Fig 7A). Another, the glycerol level of mosquitoes exposed to low temperature with water was significantly decreased at 1 h after recovery from cold exposure. However, the glycerol levels of mosquitoes exposed to subzero temperature without water had no difference with time after recovery from cold exposure. The glucose levels of mosquitoes exposed to -15 ^o^C without water were significantly increased with time after recovery from cold exposure (Fig 7B). While those exposed to -3 ^o^C directly and to -15 ^o^C with water had no difference in glucose levels with time after recovery. However, the glucose level of mosquitoes exposed to -15 ^o^C with water was significantly higher and lower than those exposed to -3 and -15 ^o^C directly at 4 h after recovery from cold exposure, respectively. These results implied that glycerol and glucose might play important role in water-induced RCH of adult mosquitoes but at different stage of cold exposure.

### Hsp70 expression in adult *Ae*. *albopictus* exposed to low subzero temperature

Because the upregulation of heat shock protein 70 (Hsp70) have been reported in several insect species in response to cold temperature [24–27], we wondered if this protein was also involved in mosquitoes to cope with cold temperature. We found that Hsp70 expression gradually increased with time after recovery from cold exposure of mosquitoes exposed to -3 ^o^C directly and to -15 ^o^C with water and the expression levels were the highest at 4 h after recovery (Fig 7C). The Hsp70 expression of mosquitoes exposed to -15 ^o^C without water was significantly increased at 1 h after recovery from cold exposure and maintained at these high levels till 4 h after recovery and the expression levels from 1 to 4 h after recovery of these mosquitoes were significantly higher than that exposed to -3 ^o^C directly and to -15 ^o^C with water. The results demonstrated that the degree of cold shock of mosquitoes exposed to -15 ^o^C with water was similar to those exposed to -3 ^o^C directly and both of them were significantly lesser than those exposed to -15 ^o^C without water.

## Discussion

Adult *Ae*. *albopictus* has long been recognized as freeze-intolerant that can’t cope with subzero temperature. Most of the studies on cold hardiness of this species was focused mainly on eggs, which were the only life stage that can survive under subzero temperature as know so far [13–19]. However, in our current study, we found that when exposed adult *Ae*. *albopictus* to low subzero temperature (-9 to -19 ^o^C) with water it could survive several hours. Moreover, LT_50_ of these adult mosquitoes were increased by 13.6 to more than 100 fold changes when compared with the counterpart exposed without water. In consistent, this phenomenon also existed in *Ae*. *aegypti* and *Cx*. *quinquefasciatus*.

Cold tolerance or cold hardiness is important for the distribution and survival of insects. Over the last 3–4 decades, *Ae*. *albopictus* has spread from its native Asian area to all continents except Antarctica [8, 9, 12]. Such widespread distribution of *Ae* .*albopictus* implied the strong cold hardiness of this species and that they might have more chance to experience sharp decrease of air temperature than other local mosquitoes. Thus far, studies about the cold hardiness of *Ae* .*albopictus* has been focused on egg stage and previous studies indicated that they overwintered predominantly through diapause eggs [16, 17, 28–30]. A previous study showed that diapause eggs from *Ae* .*albopictus* could only survive for 1 hour under -12 ^o^C while non-diapause eggs could survive for 4 hours. In addition, neither *Ae*. *albopictus* nor *Ae*. *aegypti* eggs could be hatched after exposure to -15 ^o^C [19]. However, our results showed that both the adult of *Ae*. *albopictus* and *Ae*. *aegypti* could survive under -15 ^o^C for more than 3h-exposure (Figs 1 and 2). This indicated that the cold hardiness of adult mosquitoes was even stronger than eggs when exposed with water. The cold hardiness of *Ae* .*albopictus* eggs was also highly correlated with the origin. Eggs from northern were more cold-hardy than those from southern, while eggs from tropical *Ae* .*albopictus* are much more susceptible to low temperature than those from temperate counterpart [15, 16, 18, 19]. Moreover, similar comparisons of *Ae* .*albopictus* larvae from different regions have been conducted at low temperature above zero [31]. So far as we knew, only adult *Culex pipiens* could tolerate for several to tens of hours’ exposure to subzero temperature (-5 ^o^C) [20]. Interestingly, our results showed that the cold hardiness of adult *Ae*. *albopictus*, *Ae*. *aegypti* and *Cx*. *quinquefasciatus* could be significantly enhanced in the presence of water. When exposed to -15 ^o^C with water these adult mosquitoes can survive for several hours more. This is the first report that adult *Aedes* mosquitoes cold also cope with low subzero temperature even below -10 ^o^C so long as there are waters when these mosquitoes exposed to this low temperature. Meanwhile, our results suggested that water-induced enhancement of cold hardiness might be a universal phenomenon in adult mosquitoes.

This study showed that the relative high subzero temperature of water immediate after transferring to low subzero temperature just provided partial protection for adult *Ae*. *albopictus* against low subzero temperature (Table 1). Previous studies showed that RCH of arthropods could be induced through gradual cooling from 0.1 to 1°C/min [4, 6]. We found that when transferred from room temperature to -9 to -19 ^o^C, the cooling rate of water were about 0.42 to 0.95 ^o^C/min till reaching the relatively high subzero temperature (Fig 3). These results demonstrated that RCH might be induced in these adult mosquitoes and provided partial protection during the process of cold exposure. Considering water is common in nature, our results suggested that RCH of adult mosquitoes induced by waters in nature might provide strong protection against acute decrease of air temperature to low subzero that would be lethal, which often happens in early spring and winter, and late autumn [2, 4, 7].

*Ae*. *albopictus* overwintered predominantly through diapause eggs, nevertheless, adult *Ae*. *albopictus* was also found occasionally during winter season [29, 32, 33]. This indicates these adults might experience the low subzero temperature sometimes during their lifetime like eggs do. *Ae*. *albopictus* has been considered to be the most invasive mosquito species worldwide and to be passively spread over long distance principally through the transportation of eggs by global shipments of used tires and other artificial containers [34–38]. In recent years, however, studies reported that, over a long distance, adult mosquitoes including Aedes species could be transported by aircraft [39–44], and, at a more regional level, adult *Ae*. *albopictus* are frequently transported by ground vehicles like cars [10, 37, 38]. These also pose a threat of experiencing low subzero temperature to the adult mosquitoes, especially those be transported to cooler climate areas. In the light of situations mentioned above, it is not known until now how the adult mosquitoes can cope with low subzero temperature in nature. In this study, we found that adult *Ae*. *albopictus* could cope with low subzero temperature even below -10°C in the presence of water and that after exposure to low subzero temperature for several hours the adult mosquitoes could still bite, lay eggs, and eggs could hatch to larvae (Fig 5). In addition, after a blood meal female mosquitoes must find a micro-habitat with water to lay eggs. We found that, after cold exposure to low subzero temperature with water, blood-fed *Ae*. *albopictus* could still lay viable eggs (Fig 6). This implies that water in nature not only provide a micro-habitat for mosquitoes’ egg-laying but also can act as a shelter against acute decrease of air temperature. In a word, our results indicated that the water-conferred strong protection against low subzero temperature might be an important means for adult *Ae*. *albopictus* to survive the lethal low temperature and therefore might be important for the expansion of this species to cooler areas.

Glycerol is the most commonly produced cryoprotectant for insects to cope with freeze damage [45, 46]. High accumulation in haemolymph and tissues was important for overwintering survival of insects while lower glycerol content often resulted in higher overwinter mortality [46–49]. Furthermore, the accumulation of glycerol was also highly correlated with survival of some insects in RCH [1, 3, 50–52]. In this study, we found that the glycerol levels of the adult *Ae*. *albopictus* transferred from room temperature to -15 ^o^C with water was significantly higher than those exposed to -3 and -15 ^o^C without water and those maintained at room temperature at 0h after recovery from cold exposure and then significantly decreased to normal level (Fig 7A). Because freeze damage might happen when adult mosquitoes anaesthetizing on -3 ^o^C ice and underneath -15 ^o^C air for a while, our study indicated that accumulated glycerol in adult *Ae*. *albopictus* during being exposed to -15 ^o^C with water may confer strong protection to freeze damage and contribute to RCH induced by water.

Sugars are also important cryoprotectants in insects to eliminate or minimize freeze damage [45]. There were studies that the levels of glucose in some insects were increased in response to RCH or cold stress [50, 53–55]. Our results showed that the glucose level of adult *Ae*. *albopictus* exposed to -15 ^o^C with water was significantly higher than those exposed to -3°C and lower than those exposed to -15 ^o^C without water at 4 hours after recovery from cold exposure (Fig 7B). This results indicated that the accumulation of glucose in adult *Ae*. *albopictus* was important during recovery from cold exposure but not in the process of cold exposure. Moreover, we found that glucose levels went through significant changes with time-dependent manner during recovery from cold exposure to -15 ^o^C without water. Heat shock protein 70 (Hsp70) also played an important role in cold hardiness of overwintering and cold stress of insects [27, 56, 57]. Our results showed that the expression of Hsp70 in adults *Ae*. *albopictus*, exposed to -3 or -15 ^o^C with or without water, were all significantly up-regulated during recovery from cold exposure (Fig 7C). This is consistent with previous studies of different insects (including *Culex pipiens*) that Hsp70 expression were up-regulated during recovery from exposure to subzero temperature [20, 57–59]. Our results and others indicated that the up-regulation of Hsp70 might be required for the repair of cold injury caused by cold exposure [27]. Hsp70 protein went though obvious changes when exposing adult *Ae*. *albopictus* to -15 ^o^C without water, which was similar with glucose. It increased dramatically by 6 fold from 1 to 4 h after recovery compared with those maintained under room temperature. This implied that severe acute cold shock might be happened in adult *Ae*. *albopictus* exposed to -15 ^o^C without water and caused the serious disorders of glucose metabolism that eventually lead to the death of these adult mosquitoes.

In conclusion, adult mosquitoes especially *Ae*. *albopictus* and *Ae*. *aegypti*, which are the most important vectors for dengue and Zika virus, could survive at low subzero temperature even below -10 ^o^C for several hours’ exposure in the presence of water and this cold exposure have no detriment impact on fitness costs of adult *Ae*. *albopictus*. Both the relative high subzero temperature of water immediate after cold exposure and RCH induced by gradual cooling of water provided this strong protection against low subzero temperature. The cold tolerance might be conferred by accumulation of glycerol during cold exposure stage, and contributed by both glucose accumulation and Hsp70 up-regulation during the recovery stage from cold exposure. The RCH of adult mosquitoes induced by waters in nature might provide strong protection against acute decrease of air temperature to low subzero temperature, which often happens in early spring and winter, and late autumn, and this might be important for the survival and rapid expansion of *Ae*. *albopictus* to cooler areas. Our subsequent studies would be performed further to identify whether water-induced protection could be eliminated by down-regulation of glycerol or Hsp70, and cold hardiness of eggs when exposed to low subzero temperature with water.

## Supporting information

S1 Fig The impact of cold exposure to low subzero temperature with water on the life span of adult female *Ae*. *albopictus*. Adult female *Ae*. *albopictus* were transferred from room temperature to -9 ^o^C with water for 3 h and then transferred to a cage. Survived mosquitoes were transferred to a new cage at 24h after recovery and reared under normal condition. Dead mosquitoes were counted and removed daily for a month for comparing the life span of adult female *Ae*. *albopictus* with cold exposure (blue) with those without cold exposure (red). N = 4 groups of 30 adult mosquitoes were cold exposed.
(TIF)

## References

[1] Park Y, Kim K, Kim Y. Rapid cold hardening of *Thrips palmi* (Thysanoptera: Thripidae). Environ Entomol. 2014;43(4):1076–83. 10.1603/EN13291 25182622

[2] Yang G, Wen J, Han Y, Hou M. Rapid Cold Hardening Confers a Transient Increase in Low Temperature Survival in Diapausing *Chilo suppressalis* Larvae. Insects. 2018;9(2). 10.3390/insects9020053 29747426 PMC6023533

[3] Lee RE Jr., Chen CP, Denlinger DL. A rapid cold-hardening process in insects. Science. 1987;238(4832):1415–7. 10.1126/science.238.4832.1415 17800568

[4] Ju RT, Xiao YY, Li B. Rapid cold hardening increases cold and chilling tolerances more than acclimation in the adults of the sycamore lace bug, *Corythucha ciliata* (Say) (Hemiptera: Tingidae). J Insect Physiol. 2011;57(11):1577–82. 10.1016/j.jinsphys.2011.08.012 21872604

[5] Yang S, Zhang X, Wang J, Wang S, Pan Y, Zhang J, et al Identification and analysis of up-regulated proteins in *Lissorhoptrus oryzophilus* adults for rapid cold hardening. Gene. 2018;642:9–15. 10.1016/j.gene.2017.11.002 29104168

[6] Saeidi F, Moharramipour S, Mikani A. Rapid Cold Hardening Capacity and Its Impact on Performance of Russian Wheat Aphid (Hemiptera: Aphididae). Environ Entomol. 2017;46(4):954–9. 10.1093/ee/nvx087 28541434

[7] Coleman PC, Bale JS, Hayward SA. Meat Feeding Restricts Rapid Cold Hardening Response and Increases Thermal Activity Thresholds of Adult Blow Flies, *Calliphora vicina* (Diptera: Calliphoridae). PLoS One. 2015;10(7):e0131301 10.1371/journal.pone.0131301 26196923 PMC4511429

[8] Chen XG, Jiang X, Gu J, Xu M, Wu Y, Deng Y, et al Genome sequence of the Asian Tiger mosquito, *Aedes albopictus*, reveals insights into its biology, genetics, and evolution. Proc Natl Acad Sci U S A. 2015;112(44):E5907–15. 10.1073/pnas.1516410112 26483478 PMC4640774

[9] Kotsakiozi P, Richardson JB, Pichler V, Favia G, Martins AJ, Urbanelli S, et al Population genomics of the Asian tiger mosquito, *Aedes albopictus*: insights into the recent worldwide invasion. Ecol Evol. 2017;7(23):10143–57. 10.1002/ece3.3514 29238544 PMC5723592

[10] Rudolf I, Blazejova H, Strakova P, Sebesta O, Pesko J, Mendel J, et al The invasive Asian tiger mosquito *Aedes albopictus* (Diptera: Culicidae) in the Czech Republic: Repetitive introduction events highlight the need for extended entomological surveillance. Acta Trop. 2018;185:239–41. 10.1016/j.actatropica.2018.05.020 29856987

[11] Erguler K, Smith-Unna SE, Waldock J, Proestos Y, Christophides GK, Lelieveld J, et al Large-Scale Modelling of the Environmentally-Driven Population Dynamics of Temperate *Aedes albopictus* (Skuse). PLoS One. 2016;11(2):e0149282 10.1371/journal.pone.0149282 26871447 PMC4752251

[12] Bonizzoni M, Gasperi G, Chen X, James AA. The invasive mosquito species *Aedes albopictus*: current knowledge and future perspectives. Trends Parasitol. 2013;29(9):460–8. 10.1016/j.pt.2013.07.003 23916878 PMC3777778

[13] Kress A, Oppold AM, Kuch U, Oehlmann J, Muller R. Cold tolerance of the Asian tiger mosquito *Aedes albopictus* and its response to epigenetic alterations. J Insect Physiol. 2017;99:113–21. 10.1016/j.jinsphys.2017.04.003 28396211

[14] Kress A, Kuch U, Oehlmann J, Muller R. Effects of diapause and cold acclimation on egg ultrastructure: new insights into the cold hardiness mechanisms of the Asian tiger mosquito *Aedes (Stegomyia) albopictus*. J Vector Ecol. 2016;41(1):142–50. 10.1111/jvec.12206 27232137

[15] Hanson SM, Craig GB Jr. Cold acclimation, diapause, and geographic origin affect cold hardiness in eggs of *Aedes albopictus* (Diptera: Culicidae). J Med Entomol. 1994;31(2):192–201. 8189409 10.1093/jmedent/31.2.192

[16] Hanson SM, Craig GB Jr. *Aedes albopictus* (Diptera: Culicidae) eggs: field survivorship during northern Indiana winters. J Med Entomol. 1995;32(5):599–604. 7473614 10.1093/jmedent/32.5.599

[17] Hawley WA, Pumpuni CB, Brady RH, Craig GB Jr. Overwintering survival of *Aedes albopictus* (Diptera: Culicidae) eggs in Indiana. J Med Entomol. 1989;26(2):122–9. 2709388 10.1093/jmedent/26.2.122

[18] Mogi M. Variation in cold hardiness of nondiapausing eggs of nine Aedes (Stegomyia) species (Diptera: Culicidae) from eastern Asia and Pacific islands ranging from the tropics to the cool-temperate zone. J Med Entomol. 2011;48(2):212–22. 21485356 10.1603/me10196

[19] Thomas SM, Obermayr U, Fischer D, Kreyling J, Beierkuhnlein C. Low-temperature threshold for egg survival of a post-diapause and non-diapause European aedine strain, *Aedes albopictus* (Diptera: Culicidae). Parasit Vectors. 2012;5:100 10.1186/1756-3305-5-100 22621367 PMC3403971

[20] Rinehart JP, Robich RM, Denlinger DL. Enhanced cold and desiccation tolerance in diapausing adults of *Culex pipiens*, and a role for Hsp70 in response to cold shock but not as a component of the diapause program. J Med Entomol. 2006;43(4):713–22. 10.1603/0022-2585(2006)43[713:ECADTI]2.0.CO;2 16892629

[21] Joshi D, McFadden MJ, Bevins D, Zhang F, Xi Z. Wolbachia strain wAlbB confers both fitness costs and benefit on *Anopheles stephensi*. Parasit Vectors. 2014;7:336 10.1186/1756-3305-7-336 25041943 PMC4223616

[22] Joubert DA, Walker T, Carrington LB, De Bruyne JT, Kien DH, Hoang Nle T, et al Establishment of a Wolbachia Superinfection in *Aedes aegypti* Mosquitoes as a Potential Approach for Future Resistance Management. PLoS Pathog. 2016;12(2):e1005434 10.1371/journal.ppat.1005434 26891349 PMC4758728

[23] Livak KJ, Schmittgen TD. Analysis of relative gene expression data using real-time quantitative PCR and the 2(-Delta Delta C(T)) Method. Methods. 2001;25(4):402–8. 10.1006/meth.2001.1262 11846609

[24] Huang LH, Chen B, Kang L. Impact of mild temperature hardening on thermotolerance, fecundity, and Hsp gene expression in *Liriomyza huidobrensis*. J Insect Physiol. 2007;53(12):1199–205. 10.1016/j.jinsphys.2007.06.011 17651748

[25] Sinclair BJ, Gibbs AG, Roberts SP. Gene transcription during exposure to, and recovery from, cold and desiccation stress in *Drosophila melanogaster*. Insect Mol Biol. 2007;16(4):435–43. 10.1111/j.1365-2583.2007.00739.x 17506850

[26] Huang LH, Wang CZ, Kang L. Cloning and expression of five heat shock protein genes in relation to cold hardening and development in the leafminer, *Liriomyza sativa*. J Insect Physiol. 2009;55(3):279–85. 10.1016/j.jinsphys.2008.12.004 19133268

[27] Stetina T, Kostal V, Korbelova J. The Role of Inducible Hsp70, and Other Heat Shock Proteins, in Adaptive Complex of Cold Tolerance of the Fruit Fly (*Drosophila melanogaster*). PLoS One. 2015;10(6):e0128976 10.1371/journal.pone.0128976 26034990 PMC4452724

[28] Swanson J, Lancaster M, Anderson J, Crandell M, Haramis L, Grimstad P, et al Overwintering and establishment of *Aedes albopictus* (Diptera: Culicidae) in an urban *La Crosse virus* enzootic site in Illinois. J Med Entomol. 2000;37(3):454–60. 15535592 10.1093/jmedent/37.3.454

[29] Romi R, Severini F, Toma L. Cold acclimation and overwintering of female *Aedes albopictus* in Roma. J Am Mosq Control Assoc. 2006;22(1):149–51. 10.2987/8756-971X(2006)22[149:CAAOOF]2.0.CO;2 16646341

[30] Pluskota B, Jost A, Augsten X, Stelzner L, Ferstl I, Becker N. Successful overwintering of *Aedes albopictus* in Germany. Parasitol Res. 2016;115(8):3245–7. 10.1007/s00436-016-5078-2 27112761

[31] Chang LH, Hsu EL, Teng HJ, Ho CM. Differential survival of *Aedes aegypti* and *Aedes albopictus* (Diptera: Culicidae) larvae exposed to low temperatures in Taiwan. J Med Entomol. 2007;44(2):205–10. 17427687 10.1603/0022-2585(2007)44[205:dsoaaa]2.0.co;2

[32] Tsunoda T, Chaves LF, Nguyen GT, Nguyen YT, Takagi M. Winter Activity and Diapause of *Aedes albopictus* (Diptera: Culicidae) in Hanoi, Northern Vietnam. J Med Entomol. 2015;52(6):1203–12. 10.1093/jme/tjv122 26336261

[33] Dutto M, Mosca A. Preliminary considerations about the presence of *Aedes albopictus* (Skuse 1897) (Diptera: Culicidae) during winter in the Northwestern Italy. Ann Ig. 2017;29(1):86–90. 10.7416/ai.2017.2135 28067941

[34] Hawley WA, Reiter P, Copeland RS, Pumpuni CB, Craig GB Jr. *Aedes albopictus* in North America: probable introduction in used tires from northern Asia. Science. 1987;236(4805):1114–6. 3576225 10.1126/science.3576225

[35] Tatem AJ, Hay SI, Rogers DJ. Global traffic and disease vector dispersal. Proc Natl Acad Sci U S A. 2006;103(16):6242–7. 10.1073/pnas.0508391103 16606847 PMC1435368

[36] Hofhuis A, Reimerink J, Reusken C, Scholte EJ, Boer A, Takken W, et al The hidden passenger of lucky bamboo: do imported *Aedes albopictus* mosquitoes cause dengue virus transmission in the Netherlands? Vector Borne Zoonotic Dis. 2009;9(2):217–20. 10.1089/vbz.2008.0071 18959501

[37] Flacio E, Engeler L, Tonolla M, Muller P. Spread and establishment of *Aedes albopictus* in southern Switzerland between 2003 and 2014: an analysis of oviposition data and weather conditions. Parasit Vectors. 2016;9(1):304 10.1186/s13071-016-1577-3 27229686 PMC4882898

[38] Eritja R, Palmer JRB, Roiz D, Sanpera-Calbet I, Bartumeus F. Direct Evidence of Adult *Aedes albopictus* Dispersal by Car. Sci Rep. 2017;7(1):14399 10.1038/s41598-017-12652-5 29070818 PMC5656642

[39] Eritja R, da Cunha Ramos H, Aranda C. Aircraft-mediated mosquito transport: new direct evidence. J Am Mosq Control Assoc. 2000;16(4):339 11198921

[40] Bataille A, Cunningham AA, Cedeno V, Cruz M, Eastwood G, Fonseca DM, et al Evidence for regular ongoing introductions of mosquito disease vectors into the Galapagos Islands. Proc Biol Sci. 2009;276(1674):3769–75. 10.1098/rspb.2009.0998 19675009 PMC2817279

[41] Brown EB, Adkin A, Fooks AR, Stephenson B, Medlock JM, Snary EL. Assessing the risks of West Nile virus-infected mosquitoes from transatlantic aircraft: implications for disease emergence in the United Kingdom. Vector Borne Zoonotic Dis. 2012;12(4):310–20. 10.1089/vbz.2010.0176 22217181 PMC3319934

[42] Sukehiro N, Kida N, Umezawa M, Murakami T, Arai N, Jinnai T, et al First report on invasion of yellow fever mosquito, *Aedes aegypti*, at Narita International Airport, Japan in August 2012. Jpn J Infect Dis. 2013;66(3):189–94. 23698478 10.7883/yoken.66.189

[43] Scholte EJ, Justicia AI, Stroo A, Zeeuw J, Hartog W, Reusken C. Mosquito collections on incoming intercontinental flights at Schiphol International airport, the Netherlands, 2010–2011. J Eur Mosq Control Assoc. 2014;32:17–21

[44] Mier YT-RL, Tatem AJ, Johansson MA. Mosquitoes on a plane: Disinsection will not stop the spread of vector-borne pathogens, a simulation study. PLoS Negl Trop Dis. 2017;11(7):e0005683 10.1371/journal.pntd.0005683 28672006 PMC5510898

[45] Duman JG, Wu DW, Xu L, Tursman D, Olsen TM. Adaptations of insects to subzero temperatures. Q Rev Biol. 1991;66(4):387–410

[46] Saeidi M, Moharramipour S. Physiology of Cold Hardiness, Seasonal Fluctuations, and Cryoprotectant Contents in Overwintering Adults of *Hypera postica* (Coleoptera: Curculionidae). Environ Entomol. 2017;46(4):960–6. 10.1093/ee/nvx089 28535265

[47] Izumi Y, Sonoda S, Tsumuki H. Effects of diapause and cold-acclimation on the avoidance of freezing injury in fat body tissue of the rice stem borer, *Chilo suppressalis* Walker. J Insect Physiol. 2007;53(7):685–90. 10.1016/j.jinsphys.2007.04.005 17543330

[48] Trudeau M, Mauffette Y, Rochefort S, Han E, Bauce E. Impact of host tree on forest tent caterpillar performance and offspring overwintering mortality. Environ Entomol. 2010;39(2):498–504. 10.1603/EN09139 20388280

[49] Boychuk EC, Smiley JT, Dahlhoff EP, Bernards MA, Rank NE, Sinclair BJ. Cold tolerance of the montane Sierra leaf beetle, *Chrysomela aeneicollis*. J Insect Physiol. 2015;81:157–66. 10.1016/j.jinsphys.2015.07.015 26231921

[50] Michaud MR, Denlinger DL. Shifts in the carbohydrate, polyol, and amino acid pools during rapid cold-hardening and diapause-associated cold-hardening in flesh flies (*Sarcophaga crassipalpis*): a metabolomic comparison. J Comp Physiol B. 2007;177(7):753–63. 10.1007/s00360-007-0172-5 17576567

[51] Park Y, Kim Y. RNA interference of glycerol biosynthesis suppresses rapid cold hardening of the beet armyworm, *Spodoptera exigua*. J Exp Biol. 2013;216(Pt 22):4196–203. 10.1242/jeb.092031 23948473

[52] Park Y, Kim Y. A specific glycerol kinase induces rapid cold hardening of the diamondback moth, *Plutella xylostella*. J Insect Physiol. 2014;67:56–63. 10.1016/j.jinsphys.2014.06.010 24973793

[53] Overgaard J, Malmendal A, Sorensen JG, Bundy JG, Loeschcke V, Nielsen NC, et al Metabolomic profiling of rapid cold hardening and cold shock in *Drosophila melanogaster*. J Insect Physiol. 2007;53(12):1218–32. 10.1016/j.jinsphys.2007.06.012 17662301

[54] Overgaard J, Sorensen JG, Com E, Colinet H. The rapid cold hardening response of *Drosophila melanogaster*: complex regulation across different levels of biological organization. J Insect Physiol. 2014;62:46–53. 10.1016/j.jinsphys.2014.01.009 24508557

[55] Chowanski S, Lubawy J, Spochacz M, Ewelina P, Grzegorz S, Rosinski G, et al Cold induced changes in lipid, protein and carbohydrate levels in the tropical insect *Gromphadorhina coquereliana*. Comp Biochem Physiol A Mol Integr Physiol. 2015;183:57–63. 10.1016/j.cbpa.2015.01.007 25624163

[56] Rinehart JP, Li A, Yocum GD, Robich RM, Hayward SA, Denlinger DL. Up-regulation of heat shock proteins is essential for cold survival during insect diapause. Proc Natl Acad Sci U S A. 2007;104(27):11130–7. 10.1073/pnas.0703538104 17522254 PMC2040864

[57] Colinet H, Lee SF, Hoffmann A. Temporal expression of heat shock genes during cold stress and recovery from chill coma in adult *Drosophila melanogaster*. FEBS J. 2010;277(1):174–85. 10.1111/j.1742-4658.2009.07470.x 19968716

[58] Yocum GD. Differential expression of two HSP70 transcripts in response to cold shock, thermoperiod, and adult diapause in the Colorado potato beetle. J Insect Physiol. 2001;47(10):1139–45. 12770192 10.1016/s0022-1910(01)00095-6

[59] Kostal V, Tollarova-Borovanska M. The 70 kDa heat shock protein assists during the repair of chilling injury in the insect, *Pyrrhocoris apterus*. PLoS One. 2009;4(2):e4546 10.1371/journal.pone.0004546 19229329 PMC2639642

